# Digitale Transformation deutscher Gesundheitseinrichtungen: Aktueller Stand und bestehende Herausforderungen aus Sicht des Qualitätsmanagements

**DOI:** 10.1007/s00103-023-03743-y

**Published:** 2023-08-15

**Authors:** Thomas Petzold, Oliver Steidle

**Affiliations:** 1 Medizinischer Dienst Sachsen, Dresden, Deutschland; 2 Stabsstelle Qualitätsmanagement und klinisches Risikomanagement, Universitätsklinikum Essen, Hohlweg 26, Essen, Deutschland

**Keywords:** Digitalisierung, Changemanagement, Versorgungsqualität, Gesundheitsanwendungen, Qualitätsmanagement, Digitalization, Change management, Quality of care, Health applications, Quality management

## Abstract

**Einleitung:**

Die digitale Transformation umfasst u. a. die Projektierung und Implementierung digitaler Anwendungen, Technologien und Maßnahmen und stellt inzwischen das Tagesgeschäft aller Organisationen und Einrichtungen im Gesundheitswesen dar. Im vorliegenden Beitrag werden die aktuellen Tätigkeitsinhalte der digitalen Transformation in Gesundheitseinrichtungen aus Sicht des Qualitätsmanagements dargestellt.

**Methoden:**

Es erfolgte eine deutschlandweite Befragung von 141 Mitgliedern der Gesellschaft für Qualitätsmanagement in der Gesundheitsversorgung (niedergelassene Ärztinnen und Ärzte, Vertretende von Krankenhäusern, Krankenkassen sowie weiteren Einrichtungen des Gesundheitswesens) über alle Felder der digitalen Transformation, bestehende Projekte und in der Implementierung befindliche digitale Technologien. Anhand von 6 Themenblöcken bewerteten die Teilnehmenden die zeitliche Abfolge und inhaltliche Relevanz für deren Organisation.

**Ergebnisse:**

82 % der Befragten gaben an, dass die digitale Transformation den Arbeitsalltag beeinflusst bzw. verändert. Die häufigsten Projekte umfassen Themen aus dem Prozess- und Schnittstellenmanagement (53 %) sowie zur Verbesserung der Patientensicherheit (52 %). Häufigste konkrete Vorhaben stellen die elektronische Patientenakte und Themen, die im Zuge des Krankenhauszukunftsgesetzes beauftragt wurden, dar.

**Diskussion:**

Laut Aussage der Befragten verändert die digitale Transformation Strukturen und Prozesse von Gesundheitseinrichtungen und macht zusätzliche Kompetenzen erforderlich, damit digitale Technologien zielführend und nachhaltig implementiert werden können. Eine strukturierte Koordination der Kompetenzen aller Professionen einer Gesundheitseinrichtung ist erforderlich, um digitale Technologien im Alltag von Gesundheitseinrichtungen zu verankern.

**Zusatzmaterial online:**

Zusätzliche Informationen sind in der Online-Version dieses Artikels (10.1007/s00103-023-03743-y) enthalten.

## Abstract (en)

**Introduction:**

Digital transformation includes, among other things, the project planning and implementation of digital applications, technologies, and measures and currently represents the day-to-day business of all organizations and institutions in the healthcare sector. In this article, the current activities of digital transformation in healthcare facilities from the perspective of quality management are presented.

**Methods:**

A Germany-wide survey among 141 members of the Society for Quality Management in Healthcare (physicians in private practice, hospitals, health insurers, and other healthcare institutions) was conducted on all fields of digital technologies, existing projects, and digital technologies that are currently being implemented. Based on six thematic modules, the participants evaluated the chronological sequence and the relevance of the content for their organization.

**Results:**

Of the survey participants, 82% stated that digital transformation influences or changes their everyday workplace. The most frequent projects include topics from process and interface management (53%) and improving patient safety (52%). The most frequent concrete projects are electronic patient files and topics that were commissioned as part of the *Krankenhauszukunftsgesetz*.

**Discussion:**

According to the respondents, digital transformation is changing the structures and processes of healthcare facilities and requires additional competencies so that digital technologies can be implemented in a targeted and sustainable manner. A structured coordination of the competencies of all professions in a healthcare facility is required to anchor digital technologies in the everyday life of healthcare facilities.

## Einleitung

Die digitale Transformation umfasst unter anderem die Projektierung und Implementierung digitaler Anwendungen, Technologien und Maßnahmen und stellt inzwischen das Tagesgeschäft aller Organisationen und Einrichtungen im Gesundheitswesen dar. Digitale Technologien umfassen neben der direkten Patientenversorgung auch unterstützende Prozesse der Gesundheitseinrichtungen, die nur indirekt die Patientenversorgung betreffen.

Für die Realisierung der digitalen Transformation im Gesundheitswesen müssen neben regulatorischen Voraussetzungen auch durch die jeweiligen Gesundheitseinrichtungen und Anbieter Anforderungen erfüllt werden [1]. Der Gesetzgeber hat in den letzten Jahren kontinuierlich Grundlagen geschaffen, die darauf abzielen, digitale Technologien stärker in den Versorgungskontext zu integrieren [2]. Dabei sollen „geeignete digitale medizinische Anwendungen“ [3] genutzt werden, die noch durch den Gemeinsamen Bundesausschuss zu definieren sind. Dieses Vorgehen fokussiert insbesondere auf digitale Gesundheits- bzw. Pflegeanwendungen (DiGA bzw. DiPA) und die Inhalte der Telematikinfrastruktur (TI). Sie stellen jedoch nur einen Bruchteil der digitalen Technologien dar, die durch die Gesundheitseinrichtungen projektiert und implementiert werden [2, 4–6]. In der Digitalisierungsstrategie des Bundesministeriums für Gesundheit werden diese Technologien zu einem digitalen Gesundheitsökosystem verwoben [7].

Krankenhäusern werden für die digitale Transformation finanzielle Mittel über das Krankenhauszukunftsgesetz (KHZG) und den Krankenhauszukunftsfonds bereitgestellt. Die Gesundheitseinrichtungen müssen für die digitale Transformation vor allem finanzielle Ressourcen bereitstellen. Diese Ressourcen beinhalten unweigerlich Personal- und andere Strukturmerkmale, wie bspw. das Vorhandensein:

- einer geeigneten, sicheren und belastbaren digitalen Infrastruktur,
- notwendiger und relevanter Daten (in einem geeigneten Format) für die digitale Verwendung,
- miteinander (harmonisierter und) verknüpfter Anwendungssysteme,
- ausreichenden und qualifizierten Personals für die Nutzung sowie
- einer Organisationskultur, die digitalen Technologien gegenüber aufgeschlossen ist und diese nutzen will.

Die Anforderungen an Anbieter digitaler Technologien betreffen insbesondere die Möglichkeit, relevante Daten valide verarbeiten zu können sowie die Erfassung, Verarbeitung und Ausgabe von Daten (Informationen) anwendungsfreundlich darzustellen [8]. Bei DiGA und DiPA müssen zudem die Vorgaben gemäß EU-Medizinprodukteverordnung und Medizinproduktedurchführungsgesetz gewährleistet sein. Sollten Patientinnen und Patienten oder Versicherte aktiv in die Nutzung digitaler Technologien einbezogen werden, bestehen noch zusätzliche Anforderungen [9, 10]. Aber auch semantische und syntaktische Standards sowie Interoperabilität sind durch Gesundheitseinrichtungen zu berücksichtigen und zu nutzen.

Der angestrebte Nutzen hinter diesen Anstrengungen in der Umsetzung der digitalen Transformation ist eine Verbesserung der Versorgungsqualität [11]. Erste Evaluationen lassen solche Verbesserungen vermuten [2]. Eine generelle Aussage zum Verbesserungspotenzial ist jedoch bislang nicht möglich, da jede digitale Technologie im Kontext der eingesetzten Situation gesondert zu evaluieren ist [1].

Für die Projektierung und Implementierung digitaler Technologien sind je nach Gesundheitseinrichtung unterschiedliche Berufsgruppen beteiligt, die ihre jeweiligen Kompetenzen einbringen, um zielgerichtet eine nachhaltige digitale Transformation sicherzustellen. Für die Sicherung und Weiterentwicklung der Versorgungsqualität unterstützt das Qualitäts- und Risikomanagement die jeweiligen Verantwortlichen.

Da digitale Technologien einen Einfluss auf die Versorgungsqualität haben, prüft die Gesellschaft für Qualitätsmanagement in der Gesundheitsversorgung (GQMG), welchen aktiven Beitrag das Qualitätsmanagement leisten kann, um die digitale Transformation sinnvoll mit anderen Beteiligten im Versorgungsprozess voranzutreiben, und inwiefern das Kompetenzgefüge ggf. zu erweitern ist. Um einen Überblick über die aktuellen Themen der digitalen Transformation in deutschen Gesundheitseinrichtungen zu erhalten, führte die GQMG eine Mitgliederbefragung durch.

Das Ziel der vorliegenden Arbeit ist die Darstellung aktueller Tätigkeitsinhalte des Qualitätsmanagements im Hinblick auf die digitale Transformation.

## Methoden

Die GQMG ist eine deutschlandweit agierende medizinische Fachgesellschaft, die Aufgabenfelder im Bereich des Qualitäts- und Risikomanagements in der Gesundheitsversorgung bearbeitet. Sie ist offen für alle Interessenten und zählt 459 Mitglieder (Stand: 01.04.2022). Im Zeitraum vom 28.04.2022 bis zum 20.06.2022 erfolgt eine Online-Befragung aller Mitglieder. Die Befragung wurde mit Hilfe von easy-Feedback.de durchgeführt. Alle Mitglieder wurden per E‑Mail über den Zweck, die Methodik, den Aufbau und den Umgang mit den Befragungsergebnissen informiert. Für konkrete Nachfragen standen die Geschäftsstelle der GQMG sowie die Autoren des Artikels zur Verfügung.

**Figure 1:**
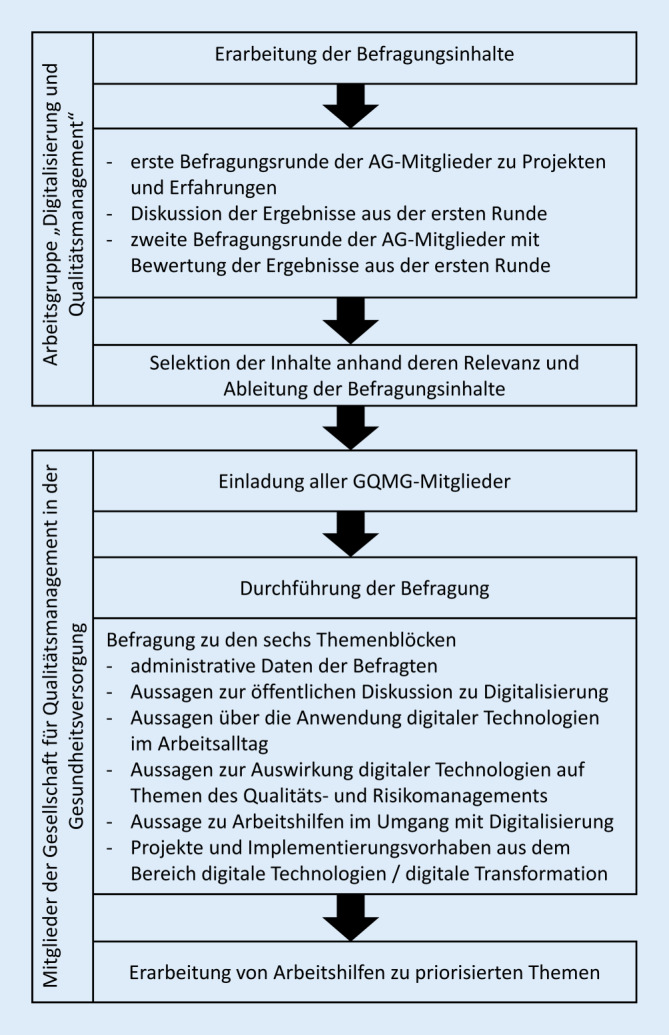

Der Mitgliederbefragung voraus ging ein Entwicklungsprozess der Befragungsinhalte in der Arbeitsgruppe „Digitalisierung und Qualitätsmanagement“ der GQMG in Form einer zweistufigen Befragung mit 2 Meetings zur Besprechung der (Zwischen‑)Ergebnisse. In der ersten Befragungsrunde konnten von den Teilnehmenden der Arbeitsgruppe (*n* = 30) aktuelle Projekte, bestehende Erfolge und Hürden aus der Projektierung und Implementierung digitaler Technologien eingebracht werden (Open-Topic-Frage). Die Ergebnisse wurde in einem ersten Meeting vorgestellt, diskutiert und in Items für die Mitgliederbefragung operationalisiert. Anschließend erfolgte eine zweite Befragungsrunde, in der alle Teilnehmenden die Relevanz der Items bewerteten. Im abschließenden, zweiten Meeting wurden diese Relevanzbewertungen vorgestellt, diskutiert und die Items nachfolgend in die Mitgliederbefragung aufgenommen (Abb. 1, [12]).

Der Fragebogen für die Mitgliederbefragung umfasste 6 Themenblöcke, in denen Fragen bzw. Aussagen zu beantworten waren (s. Onlinematerial).

- Themenblock 1: 6 administrative Fragen zu soziodemografischen Daten der Befragten
- Themenblock 2: 7 allgemeine Aussagen zu Digitalisierung
- Themenblock 3: 9 Aussagen über die Anwendung digitaler Technologien im aktuellen Arbeitsalltag
- Themenblock 4: 17 Aussagen zu Themen des Qualitäts- und Risikomanagements gemäß der Richtlinie des Gemeinsamen Bundesausschusses [13] im Hinblick darauf, ob digitale Transformation diese Themen unterstützen kann
- Themenblock 5: 13 Aussagen hinsichtlich der thematischen Erarbeitung von Arbeitshilfen für den Arbeitsalltag, ausgehend vom Qualitätsmanagement
- Themenblock 6: 5 offene Fragen zu aktuellen Digitalisierungsprojekten der Befragten

Die Aussagen der Themenblöcke 2, 4 und 5 wurden durch die Befragten anhand von 2 Dimensionen – fachliche Relevanz und zeitliche Dringlichkeit – bewertet. Die fachliche Relevanz dient der Einordnung der digitalen Technologie im Arbeitsalltag der Befragten und grenzt implizit die Tätigkeitsfelder im Qualitätsmanagement ab. Mit Hilfe der zeitlichen Dringlichkeit wurde der Stand der jeweiligen Projektierung bzw. Implementierung digitaler Technologien in den Gesundheitseinrichtungen ermittelt.

Beide Dimensionen wurden mit dem Ziel der Ableitung relevanter Handlungsfelder für die Erstellung von Arbeitshilfen der GQMG zusammengefasst. Die Befragten erfassten anhand einer Likert-Skala deren Einschätzung zu jedem Item und dessen Dimensionen – fachliche Relevanz und zeitliche Dringlichkeit. Aus allen Befragungsergebnissen wurden je Item und Dimension die arithmetischen Mittelwerte gebildet und in einem zweiachsigen Koordinatensystem visualisiert.

## Ergebnisse

Von den 459 initial zu der Befragung eingeladenen Mitgliedern der GQMG begannen 189 (41 %) mit der Beantwortung der Befragung. Insgesamt beantworteten 141 Mitglieder (31 %) die Befragung vollständig. Somit schlossen 75 % der Teilnehmenden die Befragung vollständig ab, deren Befragungsergebnisse nachfolgend präsentiert werden.

### Soziodemografische Ergebnisse

60 % (*n* = 85) der Teilnehmenden mit vollständiger Beantwortung der Befragung bezeichneten sich als weiblich, 39 % (*n* = 55) als männlich und 1 % (*n* = 1) als divers. 32 % (*n* = 46) der Befragten waren 51–60 Jahre, 28 % (*n* = 40) waren 41–50 Jahre, jeweils 17 % (*n* = 16) waren 30–40 Jahre bzw. 61–70 Jahre, 4 % (*n* = 6) waren 18–29 Jahre und 2 % (*n* = 3) 71–80 Jahre. 45 % (*n* = 64) der Befragten gaben an, Mitarbeitende im Qualitätsmanagement zu sein, 44 % (*n* = 62) eine Führungskraft im Bereich Qualitätsmanagement und 11 % (*n* = 15) im Bereich Geschäftsführung bzw. Vorstand. 73 % (*n* = 103) der Befragten nannten als Tätigkeitsort „Krankenhaus“, 16 % (*n* = 22) wählten als Tätigkeitsort „sonstiger“ und damit Institute, Beratungen oder andere Gesundheitseinrichtungen, 6 % (*n* = 9) wählten „Behörden oder Verwaltung“, jeweils 2 % (*n* = 3) wählten „Rehabilitationskliniken bzw. Alten- oder Pflegeeinrichtungen“ und 1 % (*n* = 1) „Medizinische Versorgungszentren (MVZ) oder niedergelassener Arzt“. 91 % (*n* = 128) der Befragten gaben an, dass sie im Bereich „Qualitätsmanagement“ tätig sind (= Tätigkeit) und 9 % (*n* = 13) nannten „Qualitätsmanagement“ als Funktion. 58 % (*n* = 82) der Befragten waren 11 Jahre und mehr im Bereich Qualitätsmanagement tätig, 13 % (*n* = 18) 7–10 Jahre, jeweils 12 % (*n* = 17) 3–6 Jahre bzw. 1–2 Jahre sowie 5 % (*n* = 7) weniger als 1 Jahr.

### Allgemeine Aussagen zur Digitalisierung

1. Die Digitalisierung wird mein Arbeitsumfeld im Qualitätsmanagement ganz wesentlich beeinflussen.

63 % (*n* = 88) der Befragten ordneten diese Aussage als sehr relevant ein, 33 % (*n* = 47) als relevant, 3 % (*n* = 4) als weniger relevant und 1 % (*n* = 2) als nicht relevant. 46 % (*n* = 64) der Befragten bewerteten diese Aussage als dringlich in der Umsetzung (*n* = 64), 41 % (*n* = 58) als sehr dringlich, 11 % (*n* = 16) als weniger dringlich und 2 % (*n* = 3) als nicht dringlich.

2. Die Digitalisierung wird die Kompetenzanforderungen an mich im Qualitätsmanagement ganz wesentlich beeinflussen.

45 % (*n* = 64) der Befragten ordneten diese Aussage als relevant ein, 37 % (*n* = 52) als sehr relevant, 15 % (*n* = 21) als weniger relevant und 3 % (*n* = 4) als nicht relevant. 45 % (*n* = 64) der Befragten bewerteten diese Aussage als dringlich in der Umsetzung, 30 % (*n* = 42) als sehr dringlich, 21 % (*n* = 30) als weniger dringlich und 4 % (*n* = 5) als nicht dringlich.

### Anwendung digitaler Technologien im aktuellen Arbeitsalltag

Hinsichtlich der Nutzung von Automatisierungspotenzialen in bereits bestehenden Prozessen antworten 57 % (*n* = 80) der Befragten, dass diese selten, 27 % (*n* = 38) häufig, 8 % (*n* = 11) nie und 6 % (*n* = 8) immer genutzt werden. 2 % (*n* = 4) der Befragten konnten diese Aussage nicht bewerten. Der Einsatz von Dashboards zur Bereitstellung von Reportingübersichten erfolgt nach Aussage der Befragten bei 45 % (*n* = 63) selten, 34 % (*n* = 47) häufig, 11 % (*n* = 16) nie und 9 % (*n* = 13) immer. 1 % (*n* = 2) der Befragten konnte die Aussage nicht bewerten. Bei 43 % (*n* = 61) der Befragten werden Methoden zur Datenanalyse, die den Bereichen künstliche Intelligenz oder Machine Learning zugeordnet werden, in aktuellen Prozessen nie angewendet, bei 34 % (*n* = 48) selten, 12 % (*n* = 17) häufig, 10 % (*n* = 14) konnten die Aussage nicht bewerten und 1 % (*n* = 1) sagte, dass diese Verfahren immer angewendet werden. Die Aussage, ob eine Vernetzung mit Stakeholdern zur Weitergabe von Daten erfolgt, beantworteten 44 % (*n* = 62) der Befragten mit selten, 28 % (*n* = 40) mit häufig, 13 % (*n* = 18) mit nie, 9 % (*n* = 13) konnten die Aussage nicht bewerten und 6 % (*n* = 8) antworteten mit immer. Die Berücksichtigung von Vorgaben aus Datenschutz und IT-Sicherheit berücksichtigen 47 % (*n* = 67) immer, 31 % (*n* = 43) häufig, 18 % (*n* = 25) selten; 1 % (*n* = 1) nie und 3 % (*n* = 5) konnten diese Aussage nicht bewerten. 49 % (*n* = 69) der Befragten gaben an, dass sie agile Projektstrukturen für die Projektierung digitaler Technologien selten, 23 % (*n* = 33) häufig, 19 % (*n* = 27) nie, 6 % (*n* = 8) immer im Arbeitsalltag anwenden und 3 % (*n* = 4) konnten diese Aussage nicht bewerten.

### Digitale Technologien als Unterstützung für Themen des Qualitäts- und Risikomanagements

**Figure 2:**
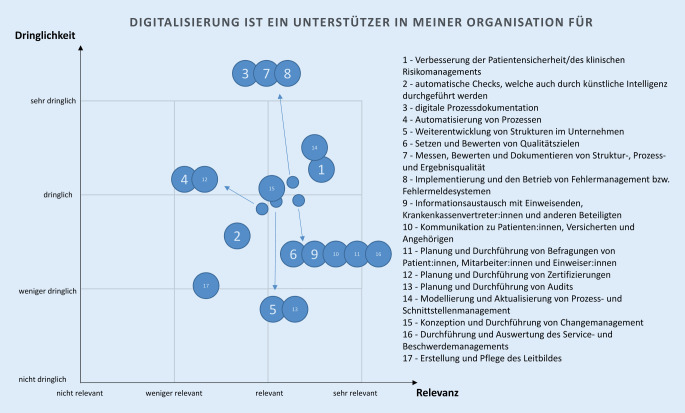

Die Befragten bewerteten die Implementierung digitaler Technologien zur Verbesserung des Prozess- und Schnittstellenmanagements als das Themenfeld des Qualitätsmanagements mit der höchsten Relevanz und Dringlichkeit. Die Implementierung digitaler Technologien für Patientensicherheit bzw. das klinische Risikomanagement, die digitale Prozessautomation, das Messen, Bewerten und Dokumentieren von Struktur‑, Prozess- und Ergebnisqualität, die Implementierung und den Betrieb von Fehlermanagement- bzw. Fehlermeldesystemen sowie die Konzeption und Durchführung des Changemanagements stuften sie als sehr relevant und sehr dringlich ein (Tab. 1). Die Implementierung digitaler Technologien für die Durchführung automatischer Checks, durch Methoden der Datenanalyse sowie für die Erstellung und Pflege des Leitbilds einer Gesundheitseinrichtung wurde als weniger relevant und weniger dringlich bewertet (Tab. 1). Die weiteren 9 Themen aus dem Bereich Qualitäts- und Risikomanagement wurden durch die Befragten als relevant und dringlich bewertet (Tab. 1, Abb. 2).

**Table 1:** 

	Fachliche Relevanz								Zeitliche Dringlichkeit							
	Sehr relevant		Relevant		Weniger relevant		Nicht relevant		Sehr dringlich		Dringlich		Weniger dringlich		Nicht dringlich	
	*n*	%	*n*	%	*n*	%	*n*	%	*n*	%	*n*	%	*n*	%	*n*	%
Erstellung und Pflege des Leitbildes	21	15	37	26	64	45	19	13	12	9	29	21	65	46	35	25
Durchführung und Auswertung des Service- und Beschwerdemanagements	56	40	73	52	12	9	0	0	40	28	56	40	38	27	7	5
Konzeption und Durchführung von Changemanagement	54	38	61	43	23	16	3	2	43	30	60	43	33	23	5	4
Modellierung und Aktualisierung von Prozess- und Schnittstellenmanagement	75	53	53	38	11	8	2	1	69	49	55	39	16	11	1	1
Planung und Durchführung von Audits	52	37	65	46	22	16	2	1	41	29	57	40	35	25	8	6
Planung und Durchführung von Zertifizierungen	39	28	63	45	33	23	6	4	31	22	51	36	45	32	14	10
Planung und Durchführung von Befragungen von Patientinnen und Patienten, Mitarbeitenden und Einweisenden	48	34	72	51	20	14	1	1	38	27	53	38	41	29	9	6
Kommunikation zu Patientinnen und Patienten, Versicherten und Angehörigen	45	32	67	48	27	19	2	1	44	31	52	37	39	28	6	4
Informationsaustausch mit Einweisenden, Krankenkassenvertretern und anderen Beteiligten	45	32	68	48	26	18	2	1	41	29	54	38	39	28	7	5
Implementierung und den Betrieb von Fehlermanagement bzw. Fehlermeldesystemen	59	42	64	45	16	11	2	1	54	38	53	38	28	20	6	4
Messen, bewerten und dokumentieren von Struktur‑, Prozess- und Ergebnisqualität	64	45	53	38	19	13	5	4	51	36	54	38	32	23	4	3
Setzen und bewerten von Qualitätszielen	55	39	55	39	27	19	4	3	40	28	58	41	36	26	7	5
Weiterentwicklung von Strukturen im Unternehmen	58	41	54	38	25	18	4	3	47	33	55	39	33	23	6	4
Automatisierung von Prozessen	37	26	66	47	35	25	3	2	29	21	63	45	46	33	3	2
Digitale Prozessdokumentation	65	46	60	43	14	10	2	1	55	39	54	38	29	21	3	2
Automatische Checks, welche auch durch künstliche Intelligenz durchgeführt werden	23	16	61	43	48	34	9	6	29	21	42	30	62	44	8	6
Verbesserung der Patientensicherheit/des klinischen Risikomanagements	74	52	54	38	12	9	1	1	62	44	59	42	17	12	3	2

### Thematische Erarbeitung von Arbeitshilfen

**Figure 3:**
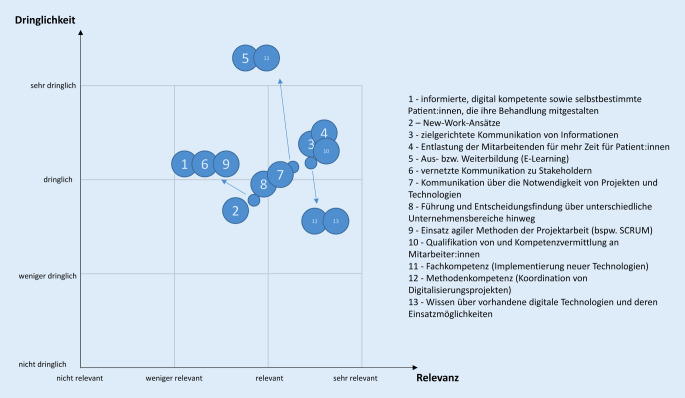

Aus Sicht der Befragten sind die Themen Entlastung der Mitarbeitenden für mehr Zeit mit Patientinnen und Patienten, zielgerichtete Kommunikation von Informationen sowie Qualifikation von und Kompetenzvermittlung an Mitarbeitende von höchster Relevanz und höchster Dringlichkeit. Hierzu sollten Arbeitshilfen im Hinblick auf Projektierung und Implementierung digitaler Technologien entworfen werden (Tab. 2). Auch als sehr relevant und sehr dringlich bewertet wurden die Themen Methodenkompetenz (Koordination von Digitalisierungsprojekten), Wissen über vorhandene digitale Technologien und deren Einsatzmöglichkeiten, Möglichkeit für Aus- und Weiterbildung (E‑Learning), Fachkompetenz (Implementierung neuer Technologien) sowie die Kommunikation über die Notwendigkeit von Projekten und Technologien (Tab. 2). Die Themen „Führung und Entscheidungsfindung über unterschiedliche Unternehmensbereiche hinweg“, „informierte, digital kompetente sowie selbstbestimmte Patientinnen und Patienten, die ihre Behandlung mitgestalten“, „vernetzte Kommunikation zu Stakeholdern“, der „Einsatz agiler Methoden der Projektarbeit“ sowie „New-Work-Ansätze“ wurden durch die Befragten als relevant und dringlich bewertet (Tab. 2, Abb. 3).

**Table 2:** 

	Fachliche Relevanz								Zeitliche Dringlichkeit							
	Sehr relevant		Relevant		Weniger relevant		Nicht relevant		Sehr dringlich		Dringlich		Weniger dringlich		Nicht dringlich	
	*n*	%	*n*	%	*n*	%	*n*	%	*n*	%	*n*	%	*n*	%	*n*	%
Informierte, digital kompetente sowie selbstbestimmte Patientinnen und Patienten, die ihre Behandlung mitgestalten	38	27	62	44	35	25	6	4	34	24	46	33	51	36	10	7
New-Work-Ansätze	27	19	68	48	33	23	13	9	25	18	54	38	48	34	14	10
Zielgerichtete Kommunikation von Informationen	79	56	56	40	6	4	0	0	66	47	60	43	14	10	1	1
Entlastung der Mitarbeitenden für mehr Zeit mit den Patientinnen und Patienten	93	66	34	24	12	9	2	1	89	63	32	23	16	11	4	3
Aus- bzw. Weiterbildung (E-Learning)	66	47	61	43	12	9	2	1	58	41	50	35	30	21	3	2
Vernetzte Kommunikation zu Stakeholdern	30	21	66	47	42	30	3	2	27	19	51	36	57	40	6	4
Kommunikation über die Notwendigkeit von Projekten und Technologien	57	40	60	43	22	16	2	1	49	35	54	38	33	23	5	4
Führung und Entscheidungsfindung über unterschiedliche Unternehmensbereiche hinweg	49	35	61	43	30	21	1	1	41	29	60	43	37	26	3	2
Einsatz agiler Methoden der Projektarbeit (bspw. SCRUM)	34	24	58	41	44	31	5	4	29	21	48	34	56	40	8	6
Qualifikation von und Kompetenzvermittlung an Mitarbeitende	79	56	52	37	10	7	0	0	66	47	59	42	13	9	3	2
Fachkompetenz (Implementierung neuer Technologien)	54	38	74	52	13	9	0	0	51	36	61	43	28	20	1	1
Methodenkompetenz (Koordination von Digitalisierungsprojekten)	69	49	59	42	13	9	0	0	65	46	51	36	23	16	2	1
Wissen über vorhandene digitale Technologien und deren Einsatzmöglichkeiten	65	46	68	48	8	6	0	0	49	35	66	47	26	18	0	0

### Aktuelle Vorhaben aus der Projektierung und Implementierung digitaler Technologien

Auf die Frage nach aktuellen Vorhaben in der Implementierung digitaler Technologien antworteten 108 Befragte. Dabei war es möglich, nicht nur ein Vorhaben, sondern unter Umständen auch mehrere Vorhaben zu benennen.

Am häufigsten wurde durch die Befragten die Patientenakte (*n* = 42) genannt. Weitere Vorhaben der Befragten sind KHZG-Projekte (*n* = 12), Medikationsmanagement (*n* = 10), Projekt- bzw. Prozessmanagement (*n* = 9), neue Internet- bzw. Intranetauftritte sowie automatisierte Feedbackmöglichkeiten (jeweils *n* = 7), der TI-Aufbau (*n* = 6), kaufmännische Prozesse, Patientendatenmanagementsysteme (PDMS), Portale für Patienteneinweiser (jeweils *n* = 5) sowie Personalmanagement, Dokumentenlenkung, Auditmanagement, klinisches Risikomanagement (jeweils *n* = 4), digitales Diktieren, Critical Incident Reporting System (CIRS), Beschwerdemanagement, E‑Learning und Krankenhausinformationssystem (KIS) (jeweils *n* = 3). Die Themen Berichtsmanagement, Gematikprojekt, Arztbriefschreibung, Dashboard, Qualitätsmanagementhandbuch, Archiv, E‑Heilberufeausweise, Entlassmanagement, Qualitätssicherung, Telemedizin sowie virtuelles Krankenhaus wurden jeweils einmal genannt.

Die Befragten konnten eine Rückmeldung geben, welche Kompetenzen aus ihrer Sicht neu hinzukommen. Darauf antworteten 41 Befragte. Sie nannten digitale Kompetenzen, Changemanagement (jeweils *n* = 10), Projekt- und Prozessmanagement, Agilität (jeweils *n* = 9), Kommunikation (*n* = 5), Umsetzungskompetenz, Führungskompetenz (jeweils *n* = 3), digitale Dokumentation (*n* = 2) sowie Anwendungskompetenz, Konfliktlösungskompetenz und Qualitätssicherung (jeweils *n* = 1).

Darüber hinaus konnten die Befragten auch mitteilen, welche Kompetenzen durch die Implementierung digitaler Technologien entfallen könnten. 40 Befragte gaben an, dass nach ihrer Einschätzung keine Kompetenzen entfallen werden. Sieben Befragte führten an, dass die Lenkung von Dokumenten, und 5, dass „Paperwork“ entfallen könnten. Jeweils ein Befragter gab an, dass Patientenrückmeldungen, Datenübermittlung und Qualitätssicherung entfallen könnten.

## Diskussion

Aus Sicht der Befragten stellen die Inhalte der digitalen Transformation sehr relevante und in der Umsetzung sehr dringliche Themen des Arbeitsalltags dar. Dennoch variieren die Angaben hinsichtlich Relevanz und Dringlichkeit zwischen den jeweiligen Themenfeldern, für welche digitale Technologien angewandt werden sollen. Besonders relevant und dringlich bewertet werden Themenfelder, die mit einem direkten Patientenkontakt assoziiert sind bzw. die direkte Maßnahmen an Patientinnen und Patienten beinhalten. Als höchst prioritär und dringlich angesehen wird die Entlastung der Mitarbeitenden für mehr Zeit für die Patientenversorgung. Dabei handelt es sich um den zentralen Tätigkeitsbereich der Gesundheitsversorgung, für den zielgerichtet und unmittelbar die Anstrengungen der digitalen Transformation im Gesundheitswesen deutlich werden sollte. Digitale Technologien werden im Tagesgeschäft aller Organisationen angewandt, nur ist der Umfang aus der Perspektive der Befragten zu gering. Aus der Befragung geht hervor, dass weitere Kompetenzen in das Qualitätsmanagement einfließen müssen, um digitale Technologien in Gesundheitseinrichtungen zu implementieren.

Auch für andere Berufsgruppen im Gesundheitswesen, die nicht unmittelbar an der Patientenversorgung teilnehmen, existieren Kompetenzanforderungen, um die digitale Transformation zielführend zu bewältigen [14–16]. Für das Qualitätsmanagement muss ersichtlich werden, welche Kompetenzen für die Realisierung der digitalen Transformation erforderlich sind, um in der Gesundheitsversorgung und der Aus- und Weiterbildung von Qualitäts- oder Risikomanagementbeauftragten relevante Inhalte zu vermitteln. Aus den Ergebnissen wird deutlich, dass die Befragten hier Nachholbedarf für das Qualitätsmanagement sehen. Für die digitale Ausgestaltung von Versorgungsstrukturen und -prozessen ist eine noch engere Zusammenarbeit zwischen fachlich Verantwortlichen und Qualitätsmanagement, IT und weiteren Bereichen erforderlich. Der stetige Austausch mit Informationssicherheits‑, Datenschutz- und IT-Sicherheitsbeauftragten wird ein Kernelement darstellen, um digitale Technologien sicher, kontinuierlich und für Patientinnen und Patienten zielführend einzusetzen.

Welche Kompetenzen für die Bewältigung der digitalen Transformation erforderlich sind, wer diese Kompetenzen ausfüllen soll und wie diese erworben werden können, wird diskutiert [14–16]. So können in bestehenden Berufsfeldern zusätzliche relevante Kompetenzen erworben [14–16] oder durch Zusatzqualifikationen erweitert werden und auch gänzlich neue Berufe entstehen [17]. Daher scheint es nicht verwunderlich, dass im Rahmen der Befragung ein Fünftel der Befragten keinen Wandel im eigenen Kompetenzprofil sah.

Mit der Einführung digitaler Technologien in Gesundheitseinrichtungen unterliegen diese Technologien den gleichen Qualitätsanforderungen, die bislang an Strukturen und Prozesse gestellt werden. Digitale Technologien sind und/oder beinhalten Prozesse, die durch geeignetes und vor allem ausreichend qualifiziertes Personal hinsichtlich deren Relevanz und Erfolg überprüft werden sollten. Diese Tätigkeiten obliegen den Verantwortlichen der jeweiligen Einsatzbereiche digitaler Technologien und können durch das Qualitätsmanagement unterstützt werden, wie es auch bereits jetzt bei der Überprüfung von Strukturen und Prozessen erfolgt. Zusätzlich können an digitale Technologien oder deren Inhalte Qualitätsanforderungen durch regulatorische Aufsichten, medizinische Fachgesellschaften oder die anwendende Gesundheitseinrichtung selbst gerichtet werden, die in geeigneter Weise überprüft werden müssen. Auch hierzu kann auf die Methoden und Kompetenzen des Qualitätsmanagements zurückgegriffen werden.

Die Ergebnisse dieser Befragung zeigen, dass sich in den Gesundheitseinrichtungen die zentralen Projekte, die auch in der Digitalstrategie thematisiert werden [7], in Bearbeitung befinden. Da mit Hilfe der digitalen Transformation nicht notwendige Prozessschritte entfallen und neue Prozessschritte mit neuen Technologien hinzukommen können, sind die Beteiligten der Gesundheitsversorgung in ausreichendem Maße zu qualifizieren. Nur so kann die Digitalisierungsstrategie [7] nachhaltig umgesetzt und ihr Transformationsansatz sichergestellt werden.

### Fragestellungen für die weitere Forschung

Anhand der Projektierung und Implementierung digitaler Technologien in Gesundheitseinrichtungen sollte überprüft werden, welche Kompetenzen fehlen bzw. zu welchen Fragestellungen Defizite in der Konzeption und Realisierung bei den Beteiligten bestehen. Diese Erhebung kann einen wertvollen Beitrag dazu leisten, das Anforderungsprofil an Beteiligte bei der Projektierung, Implementierung und dem Betrieb digitaler Technologien zu ermitteln. Mit Hilfe dieses Wissens können Gesundheitseinrichtungen personelle Ressourcen zielgerichtet planen und qualifizieren, um vor dem Hintergrund der Verdichtung von Arbeitsinhalten und Mangel an qualifiziertem Personal neue Technologien wirtschaftlich in den Arbeitsalltag einer Gesundheitseinrichtung zu integrieren. Zusätzlich sollte regelmäßig ermittelt werden, wie sich die Relevanz und Dringlichkeit bei der Implementierung digitaler Technologien verhalten. Nur mit aktuellen Statuserhebungen können Arbeitshilfen erstellt werden, die das Qualitätsmanagement dabei unterstützen, digitale Technologien zu projektieren und zu implementieren.

Falls der Nachweis von Maßnahmen des Qualitäts- und Risikomanagements nur schwer zu führen ist oder noch aussteht [18], so sollte versucht werden, für digitale Technologien diesen Nachweis methodisch valide zu führen und den Einfluss auf die Versorgungsqualität darzulegen.

### Limitationen und Stärken der vorliegenden Arbeit

Eine Schlussfolgerung aus der Rücklaufquote von 31 % und der Tatsache, dass 25 % der Teilnehmenden die Befragung nicht abgeschlossen haben, ist, dass der Fragebogen möglicherweise zu umfangreich war. Jedoch schien der initiale Aufwand erforderlich, um einen Überblick über die aktuelle Situation bei der Projektierung und Implementierung digitaler Technologien in Gesundheitseinrichtungen zu erhalten. Für zukünftige Statuserhebungen sollte ein kürzerer und fokussierter Fragebogen entwickelt werden. Eine weitere Limitation besteht im Antwortverhalten der Befragten. Möglicherweise wurden die Dimensionen (Relevanz und Dringlichkeit) für die Beantwortung der Themenblöcke 2, 4 und 5 nicht ausreichend definiert. Anhand der Abb. 2 und 3 wird deutlich, dass die Einordnung der Aussagen nahezu auf einer Geraden liegt. Dies verdeutlicht, dass die Befragten die Dimensionen nur schwer differenzieren konnten. Bei künftigen Statuserhebungen sollten die Dimensionen besser definiert werden. Eine zusätzliche Limitation liegt in der Auswahl der Aussagen. Diese waren suggestiv formuliert, um die Zustimmung und Ablehnung hinsichtlich der Relevanz und Dringlichkeit der Befragten zu ermitteln. Die Befragung stellt nur einen Ausschnitt der GQMG-Mitglieder und Qualitäts- und Risikomanagementbeauftragten in der Gesundheitsversorgung dar und wurde auf freiwilliger Basis durchgeführt. Somit kann nicht ausgeschlossen werden, dass mit mehr Teilnehmenden andere Befragungsergebnisse hätten erzielt werden können. Zusätzlich bilden die Befragungsergebnisse in großem Umfang die Einschätzung des Qualitätsmanagements in Krankenhäusern ab, da diese in der Befragung überrepräsentiert sind. Den Großteil der GQMG-Mitglieder stellen Beschäftigte aus Krankenhäusern dar, so dass diese Verteilung der Tätigkeitsorte der Befragten erwartet wurde.

Somit liegt zunächst ein breiter Überblick aller Einrichtungen vor, der organisationsbezogene Hinweise über Themen, (Miss‑)Erfolge, assoziierte Bereiche und Kompetenzen der handelnden Personen beinhaltet. Eine weitere Stärke besteht im direkten Feedback zwischen Befragten und der GQMG. So können anhand der Befragungsergebnisse Arbeitshilfen erstellt werden, die die Befragten bei der Projektierung und Implementierung digitaler Technologien unterstützen.

## Fazit

Der Großteil der Befragten gab an, dass die digitale Transformation einen Einfluss auf den Arbeitsalltag und die Kompetenzen des Qualitätsmanagements nehmen wird. Aktuell sind es vor allem Themen aus dem direkten Patientenkontakt, die als hochrelevant und dringlich eingeschätzt werden. Darunter fallen auch die Projekte, die dem KHZG zugeordnet werden, wie bspw. die Implementierung von Systemvoraussetzungen zur Dokumentation der Patient Journey zur Sicherung der Continuity of Care. Ein wesentlicher Gewinn der digitalen Transformation wird in der Entlastung der Mitarbeitenden bei der Dokumentation gesehen, aus der sich somit mehr Zeit für die eigentliche Patientenversorgung ergibt. Mit der digitalen Transformation werden neue Systeme implementiert, die neue Strukturen und Prozesse in der Erfassung und dem Management von Informationen bieten. Traditionell eher funktionell ausgerichtete Bereiche und Systeme werden aufbrechen und unterstützend tätig. Anwendenden werden damit angereicherte Informationen bereitgestellt. Entwicklungen der digitalen Transformationen benötigen daher auch eine Qualitätsplanung, -sicherung und -entwicklung.

Um digitale Technologien auch für Patientinnen und Patienten zielführend zu implementieren und nachhaltig eine Verbesserung der Versorgungsqualität zu erreichen, sollten die Kompetenzen aller Beteiligten in den Gesundheitseinrichtungen strukturiert genutzt und koordiniert werden. Das Qualitätsmanagement bietet dazu einen wertvollen Beitrag, da es bereits heute zwischen den Beteiligten der Patientenversorgung moderiert und die eigenen Kompetenzen für die digitale Transformation sinnvoll ergänzend einbringt.

## Supplementary Information




