# Supplementary material for: Digital transformation of German healthcare organizations: Current status and existing challenges from the perspective of quality management
Source: Bundesgesundheitsblatt Gesundheitsforschung Gesundheitsschutz. 2023 Aug 15;66(9):972–81. [Article in German] doi: 10.1007/s00103-023-03743-y (PMC10465675; doi:10.1007/s00103-023-03743-y)
Supplement: Supplementary file 1 [file 103_2023_3743_MOESM1_ESM.docx]

**Digitale Transformation deutscher Gesundheitseinrichtungen. Aktueller Stand und bestehende Herausforderungen aus Sicht des Qualitätsmanagements**

Thomas Petzold^1^, Oliver Steidle^2^

^1^ Medizinischer Dienst Sachsen, Dresden, Deutschland

^2^ Universitätsklinikum Essen, Stabsstelle Qualitätsmanagement und klinisches Risikomanagement, Essen, Deutschland

**Onlinematerial – Items der Befragung**

Der Fragebogen der Mitgliederbefragung umfasste sechs Themenblöcke. Jeder Themenblock wurde in Fragen bzw. Aussagen aufgegliedert, die durch die Befragten zu beantworten waren. Folgende Inhalte waren den Themenblöcken zugeordnet:

- Themenblock 1 umfasst sechs administrative Fragen zu soziodemografischen Daten der Befragten.
- Themenblock 2 beinhaltete sieben allgemeine Aussagen aus der Diskussion über Digitalisierung.
  1. Die Digitalisierung wird mein Arbeitsumfeld als handelnde Person im Qualitätsmanagement ganz wesentlich beeinflussen.
  2. Die Digitalisierung wird die Kompetenzanforderungen an mich als handelnde Person im Qualitätsmanagement ganz wesentlich beeinflussen.
  3. Mit der Digitalisierung übernehmen immer mehr Menschen Verantwortung für Ihre eigene Gesundheit.
  4. In der Zukunft wird eine ""Künstliche Intelligenz"" eine Therapie bei einer:m Patienten:in auf Basis einer automatisierten Diagnose (beispielsweise aus MRT-Bildern) vorschlagen. Die Automatisierung in der Diagnostik unterstützt dabei, beispielsweise Tumore oder Durchblutungsstörungen im Frühstadium nachzuweisen.
  5. Durch eine bessere Vernetzung aller Beteiligten in der Gesundheitsversorgung ist eine bessere Versorgung rund um die Uhr zu erwarten.
  6. Digitale Angebote, wie bspw. Gesundheits-Apps, verändern das Verordnungsverhalten von Ärzten:innen.
  7. Roboter werden mehr Aufgaben übernehmen, beispielsweise im OP oder in der Physiotherapie. Die Roboter werden einige Prozeduren, z. B. eine Naht setzen, automatisiert durchführen.
- Themenblock 3 beinhaltete neun Aussage über die Anwendung digitaler Technologien im aktuellen Arbeitsalltag
  1. Arbeitsformen (Homeoffice, mobiles Arbeiten etc.)
  2. Videokonferenzen (zoom, WebEx, GoToMeeting, BigBlueButton etc.)
  3. agile Projektstrukturen (Software, digitale Whiteboards, digitale Kanban-Boards etc.)
  4. Datenschutz und Informationssicherheit
  5. Automatisierung von Prozessen
  6. Bereitstellung von Dashboards und Reportingübersichten
  7. Nutzung von Künstlicher Intelligenz und Machine-Learning
  8. Internet der Dinge (IoT), Tagging und Tracking
  9. Vernetzung zu Stakeholdern
- Themenblock 4 umfasste 17 Aussagen zu Themen des Qualitäts- und Risikomanagements gemäß der Richtlinie des Gemeinsamen Bundesausschusses [13] im Hinblick darauf, ob die Implementierung digitaler Technologien diese Themen unterstützen kann.
  1. Verbesserung der Patientensicherheit/dem klinischen Risikomanagement
  2. automatische Checks, welche auch durch Künstliche Intelligenz durchgeführt werden
  3. digitale Prozessdokumentation
  4. Automatisierung von Prozessen
  5. Weiterentwicklung von Strukturen im Unternehmen
  6. Setzen und Bewerten von Qualitätszielen
  7. Messen, Bewerten und Dokumentieren von Struktur-, Prozess- und Ergebnisqualität
  8. Implementierung und Betrieb von Fehlermanagement bzw. Fehlermeldesystemen
  9. Informationsaustausch mit Einweisenden, Krankenkassenvertreter und anderen Beteiligten
  10. Kommunikation zu Patientinnen und Patienten, Versicherten und Angehörigen
  11. Planung und Durchführung von Befragungen von Patientinnen und Patienten, Mitarbeitenden und Einweisenden
  12. Planung und Durchführung von Zertifizierungen
  13. Planung und Durchführung von Audits
  14. Modellierung und Aktualisierung von Prozess- und Schnittstellenmanagement
  15. Konzeption und Durchführung von Changemanagement
  16. Durchführung und Auswertung des Service- und Beschwerdemanagements
  17. Erstellung und Pflege des Leitbildes
- Themenblock 5 beinhaltete 13 Aussagen hinsichtlich der thematischen Erarbeitung von Arbeitshilfen für die Projektierung und Implementierung digitaler Technologien.
  - informierte, digital kompetente sowie selbstbestimmte Patientinnen und Patienten, die Ihre Behandlung mitgestalten
  - New-Work-Ansätze
  - zielgerichteten Kommunikation von Informationen
  - Entlastung der Mitarbeitenden für mehr Zeit mit Patientinnen und Patienten
  - Aus- bzw. Weiterbildung (eLearning)
  - vernetzten Kommunikation zu Stakeholdern
  - Kommunikation über die Notwendigkeit von Projekten und Technologien
  - Führung und Entscheidungsfindung über unterschiedliche Unternehmensbereiche hinweg
  - Einsatz agiler Methoden der Projektarbeit (bspw. SCRUM)
  - Qualifikation von und Kompetenzvermittlung an Mitarbeitende
  - Fachkompetenz (Implementierung neuer Technologien)
  - Methodenkompetenz (Koordination von Digitalisierungsprojekten)
  - Wissen über vorhandene digitale Technologien und deren Einsatzmöglichkeiten
- Themenblock 6 enthielt fünf offene Fragen über aktuelle Vorhaben digitaler Technologien der Befragten.
